# Rapamycin suppresses brain aging in senescence-accelerated OXYS rats

**DOI:** 10.18632/aging.100573

**Published:** 2013-06-29

**Authors:** Nataliya G. Kolosova, Anton O. Vitovtov, Natalia A Muraleva, Andrey E. Akulov, Natalia A. Stefanova, Mikhail V. Blagosklonny

**Affiliations:** ^1^ Institute of Cytology and Genetics SB RAS, Acad. , 630090, Novosibirsk, Russia; ^2^ Department of Cell Stress Biology, Roswell Park Cancer Institute, BLSC, L3-312, Elm and Carlton Streets, Buffalo, NY, 14263, USA

**Keywords:** aging, MTOR, mTOR, brain, age-related diseases

## Abstract

Cellular and organismal aging are driven in part by the MTOR (mechanistic target of rapamycin) pathway and rapamycin extends life span in *C elegans*, *Drosophila* and mice. Herein, we investigated effects of rapamycin on brain aging in OXYS rats. Previously we found, in OXYS rats, an early development of age-associated pathological phenotypes similar to several geriatric disorders in humans, including cerebral dysfunctions. Behavioral alterations as well as learning and memory deficits develop by 3 months. Here we show that rapamycin treatment (0.1 or 0.5 mg/kg as a food mixture daily from the age of 1.5 to 3.5 months) decreased anxiety and improved locomotor and exploratory behavior in OXYS rats. In untreated OXYS rats, MRI revealed an increase of the area of hippocampus, substantial hydrocephalus and 2-fold increased area of the lateral ventricles. Rapamycin treatment prevented these abnormalities, erasing the difference between OXYS and Wistar rats (used as control). All untreated OXYS rats showed signs of neurodegeneration, manifested by loci of demyelination. Rapamycin decreased the percentage of animals with demyelination and the number of loci. Levels of Tau and phospho-Tau (T181) were increased in OXYS rats (compared with Wistar). Rapamycin significantly decreased Tau and inhibited its phosphorylation in the hippocampus of OXYS and Wistar rats. Importantly, rapamycin treatment caused a compensatory increase in levels of S6 and correspondingly levels of phospo-S6 in the frontal cortex, indicating that some downstream events were compensatory preserved, explaining the lack of toxicity. We conclude that rapamycin in low chronic doses can suppress brain aging.

## INTRODUCTION

MTOR (mechanistic Target of Rapamycin) is activated by nutrients, insulin and other hormones, growth factors, inflammatory cytokines and in turn stimulates cellular growth and functions [1-4]. In arrested cells, MTOR drives geroconversion from quiescence to senescence [5-9]. Therefore, inhibition of MTOR prolongs life span in *C elegans*, drosophila and mice [10-22].

Aging is associated with a slow deterioration of cognitive performance, particularly of learning and memory as well as with an increased risk of neurodegenerative diseases. Aging and age-associated neurodegeneration lead to behavioral impairments: impaired neuromuscular coordination and reduced exploratory activity. In a mouse model of Alzheimer's disease, rapamycin reduces amyloid-beta levels and abolishes cognitive deficits [23]. Lifelong rapamycin administration ameliorates age-dependent cognitive deficits [24]. Also, chronic treatment with rapamycin enhances learning and memory in young mice and maintains memory in old mice, and exerts anxiolytic and antidepressant-like effects [25]. Furthermore, inhibition of MTOR with everolimus (rapamycin analog) causes significant cognitive and affective improvement in humans (in heart transplant recipients) [26].

Accelerated aging in OXYS rats is associated with a reduction in locomotor and exploratory activity, greater sensitivity of old rats to anxiogenic effects of the plus-maze [27-29]. Such behavioral alterations in senescence-accelerated OXYS rats become evident by 3 months of age and are considered as manifestations the phenomenon of accelerated brain senescence [30-35]. We have shown that senescence-accelerated OXYS rats is an adequate model to study aging and age-related diseases, cerebral dysfunctions and to test anti-aging drugs to prevent age-related diseases including cognitive decline [30-35]. The behavior of young OXYS and old Wistar rats are similar. In OXYS rats, the behavioral alterations develop by the age of 3 months, preceded by progressive signs of neuro-degeneration (detected by magnetic resonance imaging, MRI) [36-39]. Another manifestation of the accelerated brain aging of OXYS rats is a decline in the ability of synapses to express plasticity and long-term potentiation (LTP) [40]. Here we investigated whether treatment with low doses of rapamycin given in food may prevent age-related brain pathology in OXY rats.

## RESULTS

### Rapamycin improved exploratory activity (rearing and head dips) in OXYS rats measured by elevated plus maze (EPM) test

**Figure 1:**
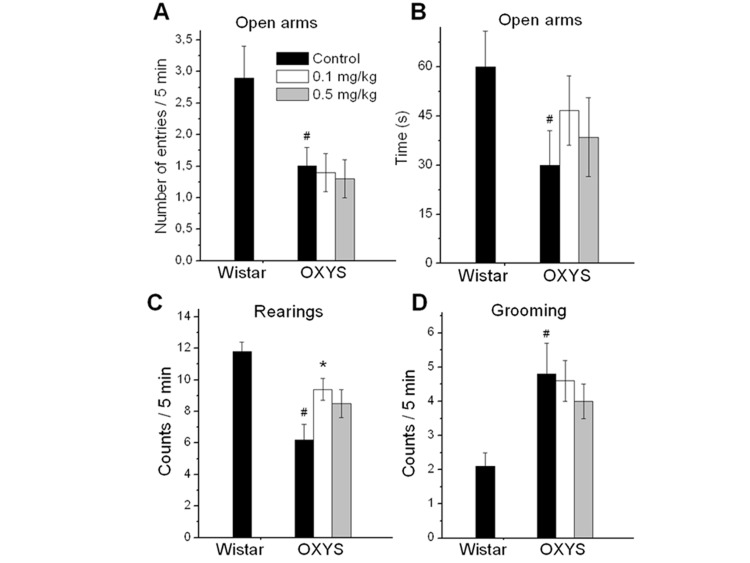
EPM performance in 3.5 month old control and rapamycin-treated OXYS rats and control Wistar rats. Treatment (0.1 or 0.5 mg/kg?day rapamycin) was started at the age of 1.5 months. OXYS rats had reduced the number of entries to open arms (**A**), time spent in open arms (**B**), frequencies of rearing (**C**), and increased grooming frequency (**D**) in comparison with Wistar rats. In rapamycin-treated OXYS rats, the of rearing was increased. Legend: ^#^p < 0.05 as the difference between the rat strains and *p < 0.05 as compared of Rapamycin-treated to the control OXYS rats.

In agreement with previous results, 3.5-month-old OXYS rats had higher scores of anxiety-like behavior compared to the age-matched Wistar rats, namely, decreased number of entries into open arms (F_1,25_ = 6.6, p < 0.02), the tendency to spend less time in open arms (F_1,25_ = 3.9, p = 0.06, Fig. 1), as well as a higher number of grooming frequency (F_1,25_ = 7.5, p < 0.02). Rapamycin treatment did not affect these indexes in OXYS rats (p < 0.05).

The number of rearings in the EPM test reflects the general exploratory activity of the animal. OXYS rats showed decreased exploratory activity compared to Wistar rats (F_1,25_ = 22.8, p < 0.0001) (Fig. 1C). Numbers of rearings in rapamycin-treated group were higher than in control group (p < 0.01 for 0.1 mg/kg rapamycin and for 0.5 mg/kg rapamycin at tendency, p=0.087). Another index of exploratory activity is the number of head dips. It was lower in control OXYS rats compared to Wistar rats (p < 0.003). The number of head dips was significantly increased in OXYS rats treated with 0.1 mg/kg rapamycin (p < 0.03).

### Rapamycin improved locomotor and exploratory activity and decreased anxiety in OXYS rats measured by open field (OF) test

**Figure 2:**
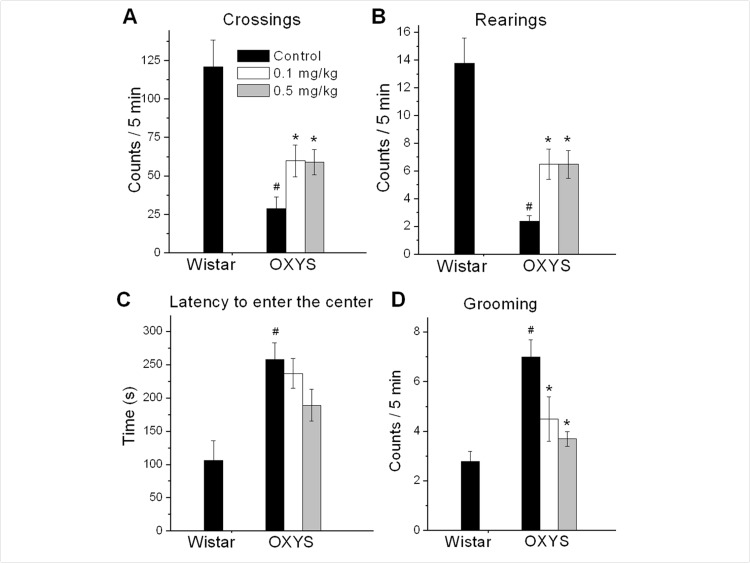
Open field performance in 3.5 month old control and rapamycin-treated OXYS rats and Wistar rats. Treatment (0.1 or 0.5 mg/kg?day rapamycin) was started at 1.5 months. OXYS rats had reduced number of squares crossed (**A**), frequencies of rearing (**B**), and increased latency time to enter the centre (**C**), grooming frequency (**D**) compared to Wistar rats. In rapamycin-treated OXYS rats the number of squares crossed, frequencies of rearing increased, latency time to enter the centre and grooming frequency were decreased. Legend: ^#^p < 0.05 as the difference between the rat strains and *p < 0.05 as compared of Rapamycin-treated to the control OXYS rats.

Open field (OF) is a versatile test that assess anxiety-like, exploratory, and locomotor behavior. As shown in figure 2, locomotor and exploratory activities (numbers line-crossings and rearings) were significantly lower in OXYS rats compared to Wistar rats (F_1,25_ = 23.3, p < 0.0001 for crossings; F_1,25_ = 37.8, p < 0.0001 for rearings). Rapamycin treatment doubled the number of line-crossing in OXYS rats (mean ± *SEM*: control, 29±7.5, 0.1 mg/kg, 60±10.3, p=0.03 and 0.5 mg/kg, 59±8.2, p=0.013, Fig. 2A) and dramatically increased the number of rearings.

The time of the first entry to the center of the OF arena and a grooming frequency are associated with anxious profiles. OXYS rats had higher levels of anxiety compared to Wistar rats (F_1,25_ = 15.5, p < 0.0007 for latency to entering the center area of the OF and F_1,25_ = 27.6, p < 0.0001 for grooming frequency). Also, rapamycin significantly decreased a grooming frequency in OXYS rats treated with 0.1 or 0.5 mg/kg rapamycin (p = 0.04 and p < 0.00, respectively).

### Rapamycin prevented brain abnormalities measured by MRI

One-way ANOVA showed that rapamycin supplementation had an impact on both relative and absolute values of cortical (F_1,10_=8.39, p<0.016 and F_1,10_=6.11, p<0.033, respectively) area. There were discovered significant interstrain differences between the relative and absolute area of hippocampus (F_1,10_=17.28, p<0.002 and F_1,10_=12.24, p<0.005; respectively) in Wistar and OXYS control groups (Table 1).

**Table 1:** Effects of rapamycin treatment (0.1 mg/kg body weight with feed from 1.5 month of age during 2 month) on parameters of the rat's brain, using MRI. (Data are presented as mean ± SEM)

Strain	Wistar	OXYS control	OXYS rapamycin
	n=6	n=6	n=6
Area of the brain, mm^2^	124.8±1.2	126.3±1.9	121.5±3.2
**Area of the cortex, mm^2^**	51.8±0.7	51.6±0.7	46.5±2.0* p<0.033 F_1,10_=6.11
Relative area “cortex/brain”, %	41.5±0.6	40.9±0.4	38.2±0.9* p<0.016 F_1,10_=8.39
Area of the hippocampus, mm^2^	10.0±1.0	13.0±0.4^#^ p<0.005 F_1,10_=12.24	12.0±0.3
Relative area “hippocampus/brain”, %	7.9±0.7	10.8±0.4^#^ p<0.002 F_1,10_=17.28	9.9±0.3
Area of the midbrain, mm^2^	36.9±1.1	40.8±0.7^#^ p<0.015 F_1,10_=8.65	37.4±2.0
Relative area “midbrain/brain”, %	29.6±0.9	32.3±0.3^#^ p<0.019 F_1,10_=7.84	30.6±0.9
Area of the lateral ventricles, mm^2^	3.31±0.94	6.37±0.40^#^ p<0.013 F_1,10_=8.98	3.17±0.57* p<0.001 F_1,10_=21.12
Relative area “lateral ventricles/brain”, %	2.633±0.73	5.045±0.31^#^ p<0.012 F_1,10_=9.28	2.632±0.49^*^ p<0.002 F_1,10_=17.12
Percentage of animals with manifestations of demyelination, %	0±0	100^#^ p<0.000 F_1,10_=49.00	50±20^*^ p<0.010 F_1,10_=10.00
The number of demyelination loci	0.00±0.00	2.33±0.33^#^ p<0.000 F_1,10_=49.00	0.50±0.34^*^ p<0.003 F_1,10_=14.76

* - a significant effect of the drug within the OXYS strain;

# - statistically significant differences between the strains of the same age;

MRI revealed hydrocephalus in control group of OXYS rats (Table 1). The area of the lateral ventricles was about 2 times greater than that of Wistar rats (F_1,10_=17.12, p<0.002 in relative and F_1,10_=21.12, p<0.001 in absolute values). Rapamycin treatment eliminated the difference between rapamycin-treated OXYS and control Wistar rats in these parameters.

We had detected no loci of demyelination in the brain of Wistar rats. At the same time, 100% of OXYS rats from control group showed signs of neurodegeneration, which was manifested by loci of demyelination. Rapamycin supplementation decreased the percentage of animals with demyelination to 50±20% (F_1,10_=10.00, p<0.010). The number of loci in control OXYS rats was 4 times higher than in rapamycin-treated animals (F_1,10_=14.76, p<0.003).

### Rapamycin decreased Tau phosphorylation in the hippocampus

**Figure 3:**
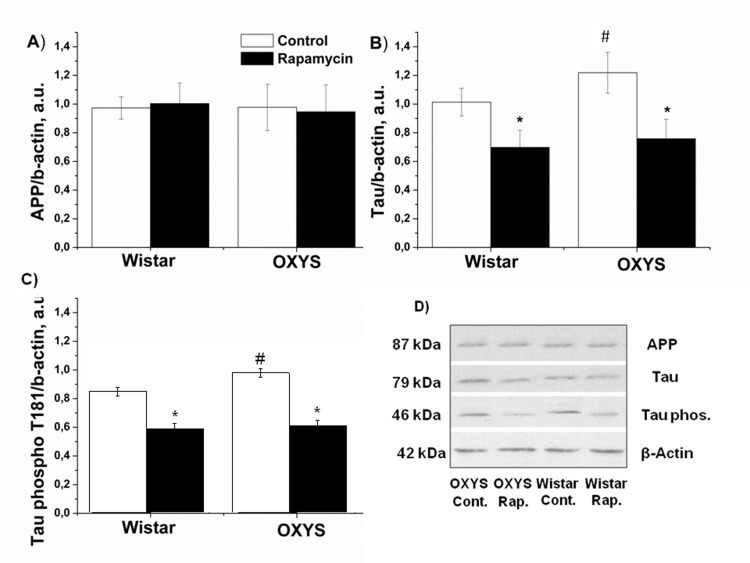
Effect of rapamycin (0.5 mg/kg per day) on Tau, Tau phospho T181 and APP in the hippocampus of OXYS and Wistar rats (n=6). **A**: Levels of App by immunoblot. **B**: Levels of total Tau. **C**: Levels of Tau phospho T181. **D**: Representative immunoblots of App, Tau and Tau phosphor T181 in the hippocampus of OXYS and Wistar rat. * -P < 0.05, statistically significant effect of rapamycin; ^#−^P < 0.05 between OXYS and Wistar rats. Data are presented as mean ± SEM.

We next studied the effect of rapamycin on the β-amyloid cascade as the target for the treatment of neurodegenerative diseases. Immunoblot analysis of soluble hippocampus homogenates revealed that the Aβ protein level in hippocampus was not affected by genotype (F_1,12_=0.52, p>0.47) or rapamicyn (F_1,12_=0.69, p<0.42). There was no difference between APP in 3-month-old OXYS and Wistar rats (Fig. 3). The level of total Tau in the rats was dependent on genotype (F_1,18_=10.2; p <0.003): in OXYS rats, it was 20 percent higher than in Wistar (p<0.003). Rapamycin significantly decreased Tau in hippocampus OXYS and Wistar rats (p<0.001 and p<0.001, respectively). Tau phosphorylation (T181) in OXYS rats was higher than in Wistar rats (p<0.017). Treatment with rapamycin induced a progressive reduction Tau phosphorylation in OXYS and Wistar rats (p<0.001 and p<0.002) (Fig. 3).

### Rapamycin increased S6 levels in the hippocampus and the frontal cortex

**Figure 4:**
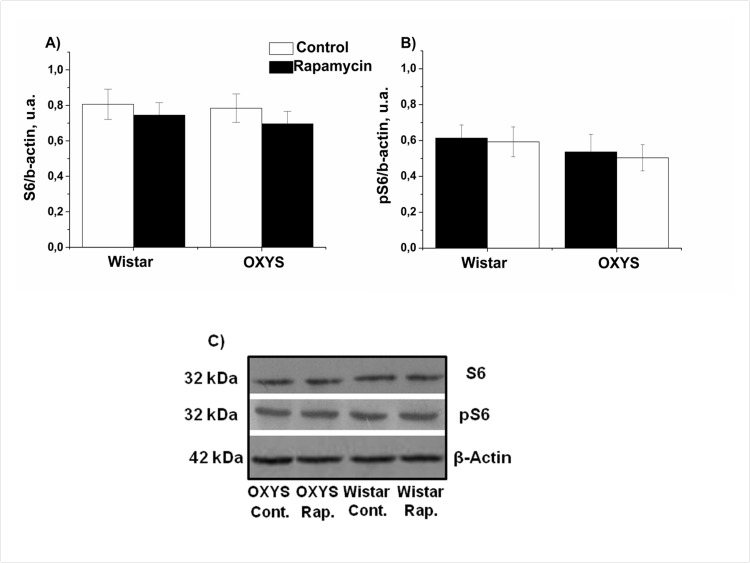
Effect of rapamycin (0.5 mg/kg per day) on the levels and phosphorylation of S6 ribosomal protein in the hippocampus of OXYS and Wistar rats (n=6) measured by immunoblot. **A**: S6. **B**: pS6. **C**: Representative immunoblots of S6 and pS6 in the hippocampus of OXYS and Wistar rats.

**Figure 5:**
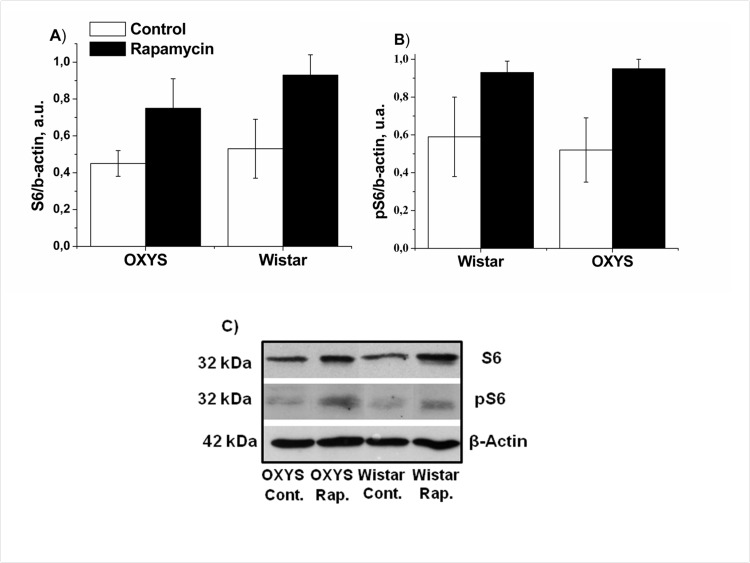
Effect of rapamycin (0.5 mg/kg per day) on the levels and phosphorylation of S6 ribosomal protein in the frontal cortex OXYS and Wistar rats (n=4) by immunoblot. **A:** S6. **B**: pS6. **C**: Representative immunoblots of S6 and pS6 in the frontal cortex of OXYS and Wistar rats.

Next we measured levels of phospho-S6, as a standard marker on MTOR activity. In control, levels of S6 and pS6 in hippocampus were similar in OXYS and Wistar rats S6 (Fig. 4). Rapamycin had no significant effect on S6 and pS6 levels (F_1,32=_0.28_,_ p<0.65 and F_1,32_ =2.31, p<0.13accordingly). Unexpectedly, we found that treatment with rapamycin increased levels of pS6 in the frontal cortex (Fig. 5). This can be explained by a corresponding increase of the S6 protein (Fig. 5 A), so that a ratio of phosphorylated and non-phosphorylated forms remained constant. This increase can be explained by compensatory reaction to chronic low doses of rapamycin, in order to spare protein synthesis. Noteworthy, some sites on S6 can be phosphorylated by other kinase pathways (such as the MEK/MAPK) pathway, independent from MTOR [41-43].

**Figure 6:**
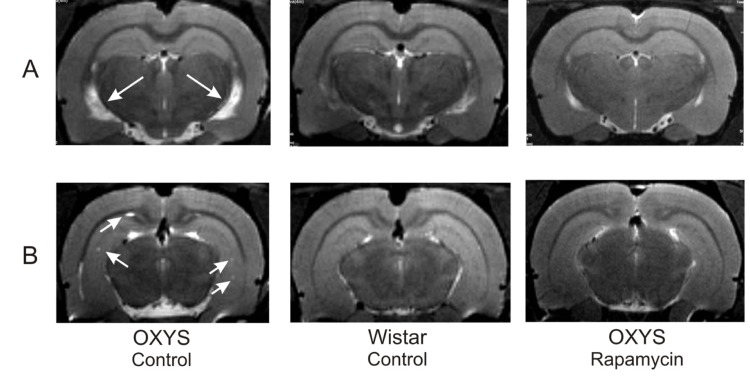
MRI image of brain 4 month-old OXYS, Wistar rats and OXYS rats after rapamycin supplementation. (**A**) Hydrocephaly of lateral ventricle of OXYS rats (arrow). (**B**) Loci of demyelinization of the brain OXYS rats (arrow).

## DISCUSSION

In agreement with our previous reports, here we described that 3-months-old OXYS rats displayed lower locomotor and exploration activities in the OF and a higher level of anxiety in the EPM tests in comparison with Wistar rats. Rapamycin treatment from the age of 1.5 months partially prevented the behavioral decline of OXYS rats. This improvement of behavior can be explained by normalization of the brain structure revealed by MRI. We demonstrated that rapamycin decreased the size of enlarged hypocampus, prevented hippocampal demyelination and decreased the volume of the lateral ventricles. Importantly, rapamycin abrogated hydrocephalus. It was shown that enlargement of ventricles is associated with accumulation of β-amyloid in the brain [44]. However we observed neither accumulation of β-amyloid nor effect of rapamycin. Instead, rapamycin decreased Tau phosphorylation, which was increased in control OXYS rats compared with Wistar rats. Noteworthy, rapamycin treatment decreased Tau phosphorylation in both rat strains. Tau is a microtubule-associated protein, which is widely expressed in neurons [45]. An increase of Tau phosphorylation and aggregation is toxic for neurons, causing nurodegeneration [46-49]. Also, tau phosphorylation impairs presynaptic function in hypertriglyceridemic mice [50].

MTOR stimulates tau phosphorylation, potentially leading to Alzheimer's disease and other tauopathies [51-54]. For example, overactivated mTOR elevates tau phosphorylation, whereas pharmacologically reducing mTOR activity with rapamycin ameliorates tau pathology and behavioral deficits [51]. Also, MTOR-mediated cell-cycle activation causes neurodegeneration in a Drosophila tauopathy model [55].

Therefore the effect of rapamycin on Tau phosphorylation provides a potential molecular mechanism for normalization of the brain structure and behavior in OXYS rats. Taken together with previous publications, our work suggests rapamycin for prevention of age-related brain degeneration and age-related diseases such as Alzheimer's disease. Rapamycin is a FDA-approved drug used in the clinic for more than a decade with tolerable side effects even at high chronic doses [56]. We did not observe side effects in OXYS rats. Yet, rapamycin is expected to decrease protein synthesis by dephosphorylating S6, a target of S6K, which is in turn a target of MTOR. Counterintuitively, we found that levels pS6 was increased in the cortex due to increased levels of the S6 protein. This may represent a compensatory reaction to chronic treatment with rapamycin at relatively low doses. This, in theory, may spare protein synthesis, even if MTOR is still inhibited. In addition, S6 can be phosphorylated via other kinase pathways, independent of MTOR [41-43]. Importantly, as shown in numerous publications, S6 phosporylation in peripheral tissues is inhibited by rapamycin, especially after acute treatment and serves as an excellent marker of the MTOR activity in cell culture.

## MATERIALS AND METHODS

### Animals, diet, treatment

All experimental procedures were in compliance with the European Communities Council Directive of 24 November 1986 (86/609/EEC). All efforts were made to minimize the number of animals used and their suffering. Male senescence-accelerated OXYS (n = 45) and age-matched male Wistar (n = 45) rats (as controls) were obtained from the Breeding Experimental Animal Laboratory of the Institute of Cytology and Genetics, Siberian Branch of the Russian Academy of Sciences (Novosibirsk, Russia). At the age of 4 weeks, the pups were weaned, housed in groups of five animals per cage (57 × 36 × 20 cm), and kept under standard laboratory conditions (22°C ± 2°C, 60% relative humidity, and natural lighting), provided with a standard rodent feed (PK-120-1, Ltd, Laboratorsnab, Russia), and given water ad libitum. 1.5-month-old OXYS and Wistar rats were randomly assigned to one of two groups (n =15): control diet or control diet supplemented with rapamycin (Rapamune; Wyeth Pharmaceuticals Inc., Berkshire, UK), 0.1 or 0.5 mg/kg of body weight per day from the age of 1.5 to 3.5 months. Body weight was measured weekly during the experiment for correction of rapamycin dose.

Rapamycin supplementation did not affect body weight in both OXYS and Wistar rats (F_2,89_ = 0.57, p > 0.59). An analysis of variance demonstrated that body weight depended on genotype (F_1,89_ =81.0, p > 0.000) and was higher in Wistar rats than in OXYS rats (at the age of 3.5 months 356 ± 32 and 296 ± 27, respectively).

### Behavioral testing

Behavioral testing began after 2 months of treatment, when rats reached the age of 3.5 months. Behavioral responses of animals to treatment were assessed in several tests in the following order: assessment of the degree of anxiety in the elevated plus-maze and observation of locomotor exploratory activity in an open field. Each test was performed once for each animal. The test sessions were conducted between 10 a.m. and 2 p.m. to avoid errors attributable to the diurnal variation of motor activity.

### Elevated plus-maze test

The plus-maze apparatus was constructed of opaque Plexiglas with two opposite open arms (50 × 10 cm) and two closed arms of the same size but with 40cm high walls. The four arms were connected by a central square (10 cm^2^) and thus formed a plus sign. The apparatus was elevated 50 cm above the floor. Each rat was placed in the central square of the plus maze facing one of the closed arms and its behavior was scored for 5 min. The number of entries with all four paws within the arms and the time spent in the arms were scored separately for open and closed arms. A greater amount of time spent in the open arms indicated reduction of anxiety-like behavior.

### Open field test

Forty eight hours after the completion of the elevated plus-maze test, the animals were subjected to the open field test, normally used to assess emotionality, based on the same conflict situation as in the EPM. The open field area consisted of an enclosed square arena made of opaque Plexiglas (100 × 100 cm) surrounded by walls (40 cm high). The arena was divided by transverse lines into 100 equal squares. A central light source (100 W) on the ceiling gave invariant illumination in an otherwise dark room. The rat to be tested was transported from the home cage to the recording room in a black box. Each rat was gently placed into the same corner of the arena facing the same direction and allowed to freely explore the arena for 5 min. Every time both hind limbs entered a square, a crossing was recorded. The locomotor and exploratory activity was evaluated from measurements of the number of line crossings and the number of rearings (the number of times the animal stood on its rear limbs). The number of grooming episodes and the time spent in the center of the field were also scored and interpreted as anxiety-like behavior.

### MRI study

All MRI experiments were performed on a horizontal 11,7T magnet (Bruker, BioSpec 117/16 USR, Germany) interfaced with a digital spectrometer operating at a resonant frequency of 500 MHz. The system is equipped with a 90-mm actively-shielded gradient set, with a maximum gradient strength of 740 mT/m. Radio frequency (RF) excitation was accomplished with a 72-mm inner diameter (ID) linear birdcage coil, and signal reception was achieved using a 45-mm ID surface coil. Each mouse was anesthetized by injecting anesthetic thiopental sodium (60 mg\kg intraperitoneal), during the procedures. The animals were placed in the prone position and then slid into the magnet bore with an animal bed. A respiratory pillow placed underneath the lower torso was used to monitor respiration (SA Instruments, Stony Brook, NY, USA). The T2-weighed images (TR = 2500 ms, TE = 11 ms, TEeff = 33 ms, number of averages: 2, RARE factor = 8; number of slices: 23, slice orientation: axial, slice thickness: 0.5 mm, inter-slice gap: 1.0 mm, field of view: 3 × 3 cm^2^, matrix: 256 × 256, scan duration: 2 min 40 s) were obtained using RARE (rapid-acquisition relaxation-enhancement) pulse sequence.

The following brain structures were investigated: hemisphere cortex; lateral ventricle; hippocampal region; midbrain and the total square of the brain. The brain structures were restricted using the manufacturer's Region of Interest (ROI) Tool software and a standard rat brain atlas by Paxinos and Watson (2007) (The rat brain in stereotaxic coordinates, Sixth Edition, Elsevier, Amsterdam). The sizes of brain structures (square, mm^2^) were calculated only in the one slice of each projection. We used coronal sections (Bregma −3.60), which were identical by the presence and relative location of brain regions among all of the animals. The foci of demyelination were visualized as hyperintense areas on the T2-weighed images, the number of which was counted in all axial sections of the brain.

### Immunoblot analysis

To measure S6 ribosomal protein and phosphorylated S6, we used hippocampus obtained from rats treated with 0.5 mg/kg rapamycin (and control animals). The hippocampus was quickly separate from the brain, placed in microcentrifuge tubes for protein isolation, and frozen in liquid nitrogen. All specimens were stored at −70°C before the analysis. Frozen tissues of hippocampus were homogenized in lysis buffer (50 mmol/L Tris-HCl, pH 7.4; 150 mmol/L NaCl; 1% Triton X-100; 1% sodium deoxycholate; 0.1% SDS and 1 mmol/L EDTA) supplemented with protease inhibitor cocktail (P8340; Sigma-Aldrich). After incubation for 20 minutes on ice, samples were centrifuged at 12.000 rpm at 4°C for 30 minutes and the supernatants were transferred to new tubes. Total proteins were measured with a Bio-Rad Bradford kit (Bio-Rad Laboratories, USA) with the bovine serum albumin as the standard. Samples were resolved on 10 % or 12% SDS-PAGE on TGB (25 mmol/L Trisbase, 190 mmol/L glycine, and 0.1% SDS) and transferred to nitrocellulose membranes (Hybon^tm^-c extra; Amersham, UK). The membranes were blocked in 5% bovine serum albumin in buffer (10 mM Tris-HCl (pH 7.5), 150 mM NaCl, 0.02% Tween-20) and probed overnight at 4 °C with following antibodies against S6 Ribosomal Protein and Phospho-S6 (Ser235/236) Ribosomal Protein (1:1000; Cell Signaling Technology, Danvers, MA). Tau, Tau (phospho T181), APP (1:1000; Abcam, USA) or β-actin (1:1000, Abcam, USA) overnight at 4 °C. The blots were developed with horseradish peroxidase-conjugated secondary anti-chiken or anti-rabbit antibodies (1:10000; Abcam, USA) and visualized with an enhanced western HRP substrate kit (Milipor, USA) and exposure to X-Ray film (Fuji, Japan). The films were scanned and the density of each of band was quantified using ImageJ (NIH, Bethesda, MD).

### Determination of Aβ protein levels in hippocampus

Enzyme-linked immunosorbent assay (ELISA) for β Amyloid (42) (Rat) ELISA Kit (Wako, Japan) was performed according to the manufacturer's instructions, except that equal protein concentrations were loaded into each well. Quantitation was carried out according to the optical density measurement obtained using a microtiter plate reader and recalculated as pg of protein β Amyloid (42) per mg of hippocampus tissue.

### Statistical analysis

The data were analyzed using repeated measures ANOVA and nonparametric tests with the statistical package Statistica 6.0. Two-way ANOVA was used to evaluate the differences between OXYS and Wistar rats across ages (age × genotype) as well as to evaluate effects of treatment (rapamycin and genotype). To test the effect of the diet on behavior parameters, the genotype and the rapamycin were chosen as independent variables. A Newman-Keuls post hoc test was applied to significant main effects and interactions in order to estimate the differences between particular sets of means. One way ANOVA was used for individual group comparisons. Data are represented as mean ± S.E.M. Comparisons between means were analyzed with one way or repeated measures analysis of variance (ANOVA), as appropriate. Results were considered statistically significant if p < 0.05.
